# Final-year medical student mental wellness during preparation for the examination for specialty in Turkey: a cross-sectional survey study

**DOI:** 10.1186/s12909-023-04063-0

**Published:** 2023-02-01

**Authors:** Mert Karabacak, Muberra Hakkoymaz, Berke Ukus, Ece Ozturk, Busra Kaya, Zeynep Ozcan, Burak Berksu Ozkara

**Affiliations:** 1 Department of Neurosurgery, Mount Sinai Health System, 1468 Madison Ave, New York, NY 10029 USA; 2 Cerrahpasa Faculty of Medicine, Istanbul University-Cerrahpasa, Cerrahpasa, Istanbul, 34098 Turkey; 3 Faculty of Medicine, Altinbas University, Mahmutbey, Istanbul, 34217 Turkey; 4 Department of Neuroradiology, MD Anderson Cancer Center, 1400 Pressler Street, Houston, TX 77030 USA

## Abstract

**Background:**

In Turkey, most final-year medical students prepare for the Examination for Specialty in Medicine in a high-stress environment. To the best of our knowledge, this is the first study on final-year medical student general psychological distress during preparation for the Examination for Specialty in Turkey. We aim to evaluate psychological distress and understand the variables associated with depression, anxiety, and stress levels among final-year medical students preparing for the Examination for Specialty.

**Methods:**

A self-reporting, anonymous, cross-sectional survey with 21 items consisting of demographic variables, custom variables directed for this study, and the DASS-21 was utilized. Survey results were expounded based on univariate analysis and multivariate linear regression analysis.

**Results:**

Our study revealed four variables associated with impaired mental wellness among final-year medical students during preparation for the examination for Specialty: attendance to preparatory courses, duration of preparation, consideration of quitting studying, and psychiatric drug usage/ongoing psychotherapy.

**Discussion:**

Considering that physician mental wellness is one of the most crucial determinants of healthcare quality, impaired mental wellness among future physicians is an obstacle to a well-functioning healthcare system. Our study targets researchers and authorities, who should focus on medical student mental wellness, and medical students themselves.

## Introduction

The World Health Organization defines wellness as “a state of complete physical, mental, and social well-being, and not merely the absence of disease or infirmity.” Poor mental wellness among physicians has consequences for both physicians as an individual and the whole healthcare system [1–3]. For instance, unwell physicians are more likely to forfeit their licenses due to medical errors and malpractice cases [4]. Research indicates a strong association between physician mental wellness and the compassionate care they provide for patients [1, 5, 6]. Predictably, a physician's productivity under stress is much lower than a healthy colleague's [7, 8]. Therefore, the importance of physician mental wellness could be considered equivalent to the importance of high-quality healthcare.

Studies on medical student mental health are imperative since medical students are future physicians, and unwell medical students may grow up as unwell physicians. Besides, medical students already have an essential role in the healthcare system as a part of their clinical education. Psychological distress among medical students is associated with profound consequences: worse academic performance, higher rates of burnout, loss of empathy, reduced quality of life, poor mental health, and suicidal ideation [9, 10]. We believe that one could forecast the future of the healthcare system by assessing medical student mental wellness and could implement novel policies to prevent further impairment.

Medical student mental wellness has attracted more attention due to studies emphasizing that most medical students endure severe psychological distress during medical school, a critical period for professional growth and personal development [11–15]. Although students' mental health profiles are average before medical school [16], they end up with significantly higher rates of burnout, depression, and other mental illnesses than their non-medical peers, indicating intense emotional consumption during medical training [1, 17–20]. According to a recent study, a significant amount of medical students and residents experience above-normal psychological distress (41.5%) or high to very high psychological distress (9.2%) [21]. that may progress into depression, anxiety, and suicidal ideation [11, 22, 23].

The most stressful aspects of medical training include preparing for exams and acquiring medical skills, which can lead to high levels of anxiety among medical students [24, 25]. Confirming the previous data, most medical students at a United States (US) medical school attributed poor wellness to the United States Medical Licensing Examination (USMLE) preparation process [26]. Thus, medical students who prepare for exams for postgraduate medical education are at stake regarding the consequences of poor mental wellness: depression, anxiety, burnout, low attention, medical errors, and negligence [14, 27, 28]. Therefore, a study on the contributors to poor mental wellness among medical students who prepare for such exams is required to comprehend previous findings; our study satisfies the need with its unique sample.

Previous studies revealed significant findings comparing first- and final-year medical students. One study found that final-year medical students had lower quality of life than first-year students, indicating a gradual decline in mental health during medical school [29]. In final-year medical students, almost all stressors are about the medical profession: medical responsibility, exams for postgraduate medical education, and less leisure time [30]. In Turkey, the preparation period for specialty examination requires intense engagement with question banks and trial tests organized by commercial preparatory courses. Besides other factors, the frequency of test-taking may also explain the higher anxiety in the final year, given that the high frequency of test-taking disrupts mental wellness [31]. In Turkey, Tıpta Uzmanlık Sınavı (Examination for Specialty in Medicine [will be mentioned as ESM from this point forward]) presumably exacerbates anxiety and stress, considering the competitive environment due to the growing number of medical schools leading to more competition among the candidates.

In Turkey, every medical student graduates as a primary care physician after six years of education. As reported by earlier studies, most of them (90%) aspire to become specialists [32]. To pursue a specialty in medicine, graduate students must pass the ESM, which assesses examinees' knowledge and understanding of basic and clinical sciences through 240 single-best-answer, multiple-choice questions. Examinees' performance rank and preferences determine the medical institutions they are assigned to for specialty training. Despite increased quotas for specialists, the ESM remains competitive due to the growing number of medical students, the accumulation of preferences in a few major branches, and the reapplication of dissatisfied specialists or failed graduates. Therefore, final-year medical students prioritize their preparation for the ESM at the cost of their mental wellness.

Although concerns about medical student mental wellness draw attention, the attempts to prevent impairment in the first place are not enough [1, 17]. The current standard for medical school education needs renovation. Unfortunately, current medical education models are limited regarding psychological training and medical skills. Regarding these, physicians are incapable of managing their mental wellness [1, 33]. In order to raise resilient physicians despite the high-stress environment, medical schools should foster student mental wellness through an adequate curriculum incorporating self-care education and coping strategies with medical training. If the factors responsible for the decline in medical student mental wellness are well-understood, authorities may develop a better educational environment with a comprehensive curriculum and psychological assistance. Therefore, we aim to understand how ESM affects mental wellness in final-year Turkish medical students by assessing their depression, anxiety, and stress levels which is a crucial aspect of mental wellness; and to pave the way for new implications targeting improved mental wellness among medical students. This study is the first to assess final-year medical students' general psychological distress by evaluating their depression, anxiety, and stress level during the preparation of ESM in Turkey.

## Methods

### Study design, setting, and participants

This cross-sectional questionnaire-based survey was conducted at Istanbul University-Cerrahpaşa, Cerrahpaşa Faculty of Medicine, in March–April 2021. Cerrahpaşa Faculty of Medicine is a public medical school in Istanbul which is one of the oldest and most prestigious medical schools in Turkey. The English program typically accepts between 80 and 100 students per year, while the Turkish program accepts between 250 and 300 students annually while it is subject to change. Medical students in the final year of their medical education were targeted. The first three years of the medical school program at the Cerrahpaşa Faculty of Medicine are preclinical, while the fourth and fifth years are when the clinical clerkships are done. The sixth year, the final year, is a year of pre-graduate internship. In their final year, medical students serve as full-time intern doctors in the Cerrahpaşa Faculty of Medicine Hospitals. In addition to rotations in major branches, such as the departments of internal medicine, general surgery, pediatrics, emergency medicine, gynecology, and obstetrics, they rotate in minor branches. They do not take exams at the institution, but their full-time internship with regular night shifts makes it difficult for them to study for ESM. Thus, the participants of this study were sixth-year medical students. An online survey was hosted on SurveyMonkey (www.surveymonkey.com). It was distributed to all eligible students through WhatsApp and Telegram text messages, owing to a lack of a proper students' email database. Two reminder text messages were also sent out ten days apart. A link to the survey and detailed instructions were included in the text messages. Before starting the survey, participants were provided a summary of the study, explaining its purpose, its scientific and academic nature, terms of anonymity, and confidentiality. Participants consented to their anonymized data being used by checking a box. This study was reviewed and approved by the institutional review board of the Cerrahpaşa Faculty of Medicine.

### Questionnaire

A self-reporting, anonymous, cross-sectional survey was conducted using the Depression, Anxiety, and Stress Scale (DASS-21) and also 21 custom questions regarding demographic, educational, and custom variables designed for this study [34]. Nine demographic and educational variables consisted of age, sex, the language of education, grade point average (GPA), relationship status, socioeconomic status, duration of preparation, weekly study hours, and attendance to ESM preparatory courses.

Additional twelve questions that can be related to mental wellness were asked. Three of them were related to the perceived status of their study: (1) whether they consider their study sufficient, (2) think that they get the worth of their study, and (3) consider quitting studying or not. Furthermore, one question regarding their reflection on working abroad was present. Psychiatric drugs and prescription stimulants usage were evaluated, as well as cigarette, alcohol, or drug consumption. The remaining questions were about setting aside time for themselves and family or friends, mindfulness to nutrition, sleep sufficiency, and exercising.

The DASS-21 is a 21-item self-assessment tool for healthcare providers. It had good internal consistency (all a > 0.80), test–retest reliability (all Mutual Information Scores > 0.80), and content and discriminant validity in the Turkish adaptation study of the scale [35]. All DASS-21 items are on a seven-point Likert scale (1 = strongly disagree, 7 = strongly agree). Eight of the 18 items are reverse-scored. Each subscale is made up of the averages of select items (eight "Depression", nine "Anxiety", and fourteen "Stress" items). The overall mean score ("Overall DASS-21") is made up of the averages of all 21 items. Higher scores indicate greater levels of depression, anxiety, and stress, which implies reduced mental wellness.

### Statistical analysis

We calculated frequencies for categorical variables, means with standard deviations (SD) for normally distributed continuous variables, and medians with interquartile ranges (IQR) for non-normally distributed continuous variables. Spearman's rank correlation was conducted to assess the relationship between the only continuous variable age and dependent variables, DASS-21, and its subscales' mean scores. For categorical variables with two groups, we performed an independent t-test for normally distributed dependent variables with equal variances, Welch's t-test for normally distributed dependent variables with unequal variances, and Mann–Whitney U test for non-normally distributed dependent variables. For categorical variables with three or more groups, one-way analysis of variance (ANOVA) for normally distributed dependent variables and the Kruskal Wallis test for non-normally distributed dependent variables were performed. Variables with a p-value smaller than 0.2 in the univariate analysis were used in the multivariate linear regression models. A p-value of 0.05 was considered to indicate statistical significance. All statistical analyses were performed in R 4.1.3 [36].

## Results

### Demographic findings

A total of 146 final-year medical students participated in this study; 101 responses were complete submissions and used in the analysis yielding a response rate of 21.35% (101/473). The mean age was 23.97 (SD = 1.27), and it was not significantly associated with the DASS-21 mean score and any of the three subscales' mean scores. Of the 101 participants, 7.92% reported GPA between 2.0–2.5, 38.61% between 2.5–3.0, 33.66% between 3.0–3.5, and 19.80% between 3.5–4.0. Regarding the duration of preparation, most respondents (*n* = 62, 61.39%) spent 1–2 years, 7.92% spent more than 2 years, 11.88% spent 6 months-1 year, and 18.81% spent less than 6 months. Less than half of the respondents (*n* = 39, 38.61%) spent 30–60 h a week. Among others, 14.85% spent more than 60 h, 15.84% spent 15–30 h, and 30.69% spent less than 15 h. A substantial majority (*n* = 92, 91.09%) of the participants attended a preparatory course.

About half (*n* = 53, 52.48%) of the students considered their study insufficient, a little more than a quarter (*n* = 27, 26.73%) considered their study sufficient, and 20.79% were unsure regarding the sufficiency of their study. Although 29.70% never considered quitting studying, more than two third of students considered quitting studying (38.61% rarely, 20.79% often, 10.89% always). 34.65% of students used prescription psychiatric drugs or undergone psychotherapy, 31.68% used prescription stimulants to increase productivity, and 46.53% started using or increased the consumption of cigarettes, alcohol, or drugs during preparation for ESM. Most students were not mindful of their nutrition, did not get enough sleep, and did not set aside time for exercising. These variables and their association with the subscale scores are given in Table 1.

**Table 1:** Descriptive statistics and subscale scores according to variables

			**DASS-21 Depression**		**DASS-21 Anxiety**		**DASS-21 Stress**	
**Variables**	***n***	**% or Mean (SD)**	**Mean (SD)**	***p***	**Mean (SD)**	***p***	**Mean (SD)**	***p***
**Age**	-	23.97 (1.27)	-	0.365	-	0.829	-	0.374
**Sex**								
Male	50	49.50%	13.88 (8.37)	0.445	9.32 (7.20)	0.174	14.28 (8.43)	0.160
Female	51	50.50%	15.22 (8.88)		11.49 (7.66)		16.71 (7.83)	
**Language of education**								
Turkish	32	31.68%	14.61 (8.65)	0.886	10.99 (7.54)	0.251	15.83 (8.59)	0.840
English	69	68.32%	14.44 (8.67)		9.19 (7.32)		14.81 (7.29)	
**GPA**								
2.0—2.5	8	7.92%	17 (9.62)	0.089	11.75 (6.27)	0.076	15.00 (9.26)	0.121
2.5—3.0	39	38.61%	16.21 (9.14)		11.64 (7.00)		17.74 (7.83)	
3.0—3.5	34	33.66%	11.82 (8.27)		7.88 (7.15)		13.65 (9.19)	
3.5—4.0	20	19.80%	15 (7)		11.80 (8.70)		14.50 (5.80)	
**Relationship status**								
Not in a relationship	57	56.44%	15.33 (9.74)	0.419	10.91 (6.91)	0.283	15.33 (9.07)	0.522
In a relationship	44	43.56%	13.55 (7.36)		9.77 (8.20)		15.73 (6.96)	
**Socioeconomic status**								
Low/Lower-middle	24	23.76%	17.58 (8.21)	0.085	9.50 (6.47)	0.661	13.92 (7.81)	0.157
Middle	46	45.54%	14.13 (8.05)		10.09 (7.33)		15.04 (8.11)	
Upper/Upper-middle	31	30.69%	12.84 (9.38)		11.61 (8.46)		17.42 (8.46)	
**Duration of preparation**								
More than 2 years	8	7.92%	13.25 (8.75)	0.846	10.50 (6.21)	0.490	18.75 (8.35)	**0.013**
1–2 years	62	61.39%	15.23 (9.21)		10.32 (7.88)		16.07 (8.02)	
6 months-1 year	12	11.88%	13.17 (6.12)		7.83 (5.29)		9.00 (4.71)	
Less than 6 months	19	18.81%	13.79 (8.24)		12.32 (7.75)		16.42 (8.83)	
**Weekly study hours**								
More than 60 h	15	14.85%	12 (6.05)	0.286	8.27 (7.32)	0.365	16.13 (5.37)	0.779
30–60 h	39	38.61%	14.46 (9.8)		9.85 (7.76)		15.90 (9.65)	
15–30 h	16	15.84%	17.38 (7.03)		11.13 (6.49)		13.50 (5.14)	
Less than 15 h	31	30.69%	14.45 (8.74)		11.81 (7.68)		15.74 (8.71)	
**Attendance to preparatory course**								
Yes	92	91.09%	15.13 (8.66)	**0.026**	10.78 (7.62)	0.133	16.02 (7.99)	**0.041**
No	9	8.91%	8.67 (5.66)		6.67 (4.69)		10.22 (8.69)	
**"Do you think that your study is sufficient?"**								
Yes	27	26.73%	11.48 (6.6)	0.152	8.44 (7.65)	0.207	13.56 (5.91)	0.568
No	53	52.48%	15.43 (8.65)		11.36 (7.73)		16.08 (8.38)	
Unsure	21	20.79%	16.29 (9.64)		10.57 (6.36)		16.57 (9.98)	
**"Do you think that get the worth of your study?"**								
Yes	37	36.63%	14.05 (7.85)	0.973	11.35 (7.30)	0.535	15.24 (7.32)	0.110
No	38	37.62%	15.11 (9.61)		10.11 (8.23)		17.47 (8.24)	
Unsure	26	25.74%	14.46 (8.42)		9.54 (6.68)		13.00 (8.80)	
**"Do you consider quit studying?"**								
Never	30	29.70%	10.73 (6.4)	**0.005**	6.07 (6.38)	** < 0.001**	13.20 (6.98)	0.037
Rarely	39	38.61%	14.46 (7.94)		10.77 (7.04)		14.51 (7.63)	
Often	21	20.79%	16.1 (9.09)		12.19 (6.03)		18.38 (9.44)	
Always	11	10.89%	22.36 (10.15)		17.64 (7.68)		19.82 (8.41)	
**"Would you like to work abroad in the future if you have chance?"**								
Yes	74	73.27%	14.08 (8.92)	0.228	10.35 (6.63)	0.172	15.76 (8.10)	0.620
No	13	12.87%	14.31 (9.2)		8.15 (9.64)		15.23 (9.33)	
Unsure	14	13.86%	17.29 (6.11)		12.86 (9.27)		14.43 (8.01)	
**"Have you used prescription psychiatric drugs (antidepressants, etc.) or undergone psychotherapy durıng your ESM preparation?"**								
Yes	35	34.65%	15.89 (8.12)	0.138	13.09 (7.30)	**0.007**	18.97 (6.60)	** < 0.001**
No	66	65.35%	13.85 (8.85)		9.00 (7.23)		13.67 (8.38)	
**"Have you used prescription stimulants (amphetamines, etc.) to increase your productivity durıng your ESM preparation?"**								
Yes	32	31.68%	14.5 (8.05)	0.858	12.17 (8.29)	0.438	21.33 (6.51)	**0.004**
No	69	68.32%	14.56 (8.73)		10.18 (7.38)		14.72 (8.09)	
**"Can you set aside time for yourself apart from studying?"**								
Yes	51	50.50%	13.73 (8.41)	0.270	9.41 (7.24)	0.176	13.29 (7.03)	**0.008**
No	50	49.50%	15.4 (8.83)		11.44 (7.66)		17.76 (8.71)	
**"Can you set aside time for your friends and family?"**								
Yes	47	46.53%	13.62 (8.34)	0.279	9.19 (7.35)	0.113	13.19 (7.49)	**0.004**
No	54	53.47%	15.37 (8.85)		11.48 (7.50)		17.52 (8.29)	
**"Have you started using or increased your consumption of cigarettes, alcohol or drugs during your ESM preparation?"**								
Yes	47	46.53%	14.13 (6.09)	0.907	11.44 (7.10)	0.271	18.50 (6.76)	**0.004**
No	54	53.47%	14.75 (9.6)		9.94 (7.65)		14.12 (8.45)	
**"Can you be mindful of your nutrition?"**								
Yes	38	37.62%	12.63 (7.84)	0.106	9.89 (7.08)	0.642	15.00 (7.54)	0.693
No	63	62.38%	15.71 (8.91)		10.73 (7.75)		15.81 (8.59)	
**"Can you get enough sleep?"**								
Yes	36	35.64%	13.56 (9.39)	0.281	8.28 (6.68)	**0.039**	13.89 (7.50)	0.119
No	65	64.36%	15.11 (8.18)		11.60 (7.68)		16.40 (8.46)	
**"Can you set aside time for exercising"**								
Yes	20	19.80%	11.8 (8.08)	0.099	9.00 (7.36)	0.338	13.60 (7.86)	0.194
No	81	80.20%	15.23 (8.66)		10.77 (7.51)		15.98 (8.24)	

### DASS-21 depression subscale

The attitude subscale showed high internal reliability (a = 0.84) with a mean score of 10.42 (7.48). In the univariate analysis (Table 1). attendance to preparatory courses (*p* = 0.026) and consideration of quitting studying (*p* = 0.005) were found to be statistically significant. The multivariate regression model (Table 2). showed that those who did not attend preparatory courses received lower scores than those who attended preparatory courses by 5.82 points [*p* = 0.046, coefficient = -5.82, 95% confidence interval [CI] = (-11.52) – (-0.10)]. Participants who never [*p* = 0.001, coefficient = -10.13, 95% CI (-15.82)—(-4.44)], rarely [*p* = 0.006, coefficient = -8.03, 95% CI (-13.73)—(-2.34)], or often [*p* = 0.029, coefficient = -6.87, 95% CI (-13.00)—(-0.73)] considered quitting studying had lower scores than those who stated always considering quit studying.

**Table 2:** Multivariate linear regression results

	**DASS-21 Depression**		**DASS-21 Anxiety**		**DASS-21 Stress**	
**Variables**	**Coefficient (95% CI)**	***p***	**Coefficient (95% CI)**	***p***	**Coefficient (95% CI)**	***p***
**Sex**						
Male	-		Reference		Reference	
Female			1.74 (-1.25—4.73)	0.250	2.26 (-0.75—5.27)	0.139
**GPA**						
2.0—2.5	Reference		Reference		Reference	
2.5—3.0	-3.47 (-10.03—3.08)	0.295	-0.62 (-5.91—4.67)	0.250	-1.63 (-7.34—4.08)	0.572
3.0—3.5	-4.81 (-11.36—1.75)	0.149	-2.39 (-7.79—3.00)	0.817	-2.81 (-8.62—2.99)	0.338
3.5—4.0	-3.77 (-10.80—3.27)	0.291	-2.07 (-8.00—3.87)	0.380	-0.93 (-7.19—5.33)	0.768
**Socioeconomic status**						
Low/Lower-middle	Reference		-		Reference	
Middle	-2.25 (-6.31—1.81)	0.273			2.25 (-1.45—5.96)	0.230
Upper/Upper-middle	-2.56 (-7.17—2.04)	0.272			2.11 (-2.22—6.44)	0.335
**Duration of preparation**						
More than 2 years	-		-		-0.66 (-6.18—4.87)	0.813
1–2 years					Reference	
6 months-1 year					-1.19 (-5.94—3.55)	0.619
Less than 6 months					5.19 (1.00—9.38)	**0.016**
**Attendance to preparatory course**						
Yes	Reference		Reference		Reference	
No	-5.82 (-11.53—-0.11)	**0.046**	-1.66 (-6.65—3.33)	0.511	-2.83 (-8.01—2.35)	0.280
**"Do you think that your study is sufficient?"**						
Yes	Reference		-		-	
No	3.14 (-0.92—7.20)	0.128				
Unsure	4.10 (-0.89—9.08)	0.106				
**"Do you think that get the worth of your study?"**						
Yes	-		-		Reference	
No					-2.10 (-5.58—1.39)	0.234
Unsure					-2.54 (-6.23—1.15)	0.175
**"Do you consider quit studying?"**						
Never	-10.13 (-15.83—-4.44)	**0.001**	-10.65 (-15.41—-5.89)	**0.000**	-10.95 (-15.95—-5.95)	**0.000**
Rarely	-8.03 (-13.73—-2.34)	**0.006**	-6.04 (-10.95—-1.12)	**0.017**	-6.62 (-12.00—-1.24)	**0.016**
Often	-6.87 (-13.00—-0.73)	**0.029**	-3.85 (-9.22—1.52)	0.157	-7.08 (-12.49—-1.67)	**0.011**
Always	Reference		Reference		Reference	
**"Would you like to work abroad in the future if you have chance?"**						
Yes	-		Reference		-	
No			0.58 (-4.00—5.17)	0.801		
Unsure			3.24 (-1.34—7.83)	0.163		
**"Have you used prescription psychiatric drugs (antidepressants, etc.) or undergone psychotherapy durıng your TUS preparation?"**						
Yes	Reference		Reference		Reference	
No	-1.97 (-5.48—1.55)	0.270	-3.35 (-6.38—-0.33)	**0.030**	-2.74 (-6.35—0.86)	0.134
**"Have you used prescription stimulants (amphetamines, etc.) to increase your productivity durıng your TUS preparation?"**						
Yes	-		-		Reference	
No					-1.41 (-6.54—3.73)	0.586
**"Can you set aside time for yourself apart from studying?"**						
Yes	-		Reference		Reference	
No			1.40 (-2.10—4.91)	0.429	2.20 (-1.70—6.10)	0.264
**"Can you set aside time for your friends and family?"**						
Yes	-		Reference		Reference	
No			-0.54 (-4.02—2.94)	0.757	-0.40 (-4.27—3.46)	0.836
**"Have you started using or increased your consumption of cigarettes, alcohol or drugs during your TUS preparation?"**						
Yes	-		-		Reference	
No					0.62 (-3.22—4.45)	0.751
**"Can you be mindful of your nutrition?"**						
Yes	Reference		-		-	
No	3.35 (-0.23—6.94)	0.066				
**"Can you get enough sleep?"**						
Yes	-		Reference		Reference	
No			2.02 (-1.30—5.35)	0.230	3.93 (0.42—7.44)	**0.029**
**"Can you set aside time for exercising"**						
Yes	Reference		-		Reference	
No	2.61 (-1.70—6.91)	0.232			-0.22 (-4.12—3.69)	0.913

### DASS-21 anxiety subscale

The anxiety subscale was internally reliable (a = 0.81), with a mean score of 14.55 (8.62). The univariate analysis (Table 1) revealed that participants who considered quitting studying (*p* < 0.001), used prescription psychiatric drugs, or underwent psychotherapy (*p* = 0.007), and were deprived of sufficient sleep (*p* = 0.039) had higher anxiety levels than others. In multivariate analysis (Table 2), respondents who always considered quitting studying had higher scores than those who never [*p* < 0.001, coefficient = -10.65, 95% CI (-15.41) – (-5.89)] or rarely [*p* = 0.017, coefficient = -6.04, 95% CI (-10.95) – (-1.12)] considered quitting studying. Lastly, medical students using prescription psychiatric drugs or receiving psychotherapy during the preparation for ESM had higher scores than those who did not [*p* = 0.03, coefficient = -3.35, 95% CI (-6.38) – (-0.33)].

### DASS-21 stress subscale

The stress subscale showed high internal reliability (a = 0.86) with a mean score of 15.50 (8.18). In the univariate analysis (Table 1), we found statistical significance in the mean subscale scores for eight variables: duration of preparation (*p* = 0.013), attendance to a preparatory course (*p* = 0.041), consideration of quitting studying (*p* = 0.037), prescription psychiatric drugs usage or ongoing psychotherapy (*p* < 0.001), prescription stimulants usage (*p* = 0.004), setting aside time for oneself (*p* = 0.008), setting aside time for family or friends (*p* = 0.004), and having started to use or increased consumption of cigarettes, alcohol, or drugs (*p* = 0.004). The multivariate model (Table 2). showed that those who prepared for less than 6 months (*p* = 0.016, coefficient = 5.19, 95% CI 1.00 – 9.38) were more depressed than those who prepared for 1–2 years. Final year medical students who never [*p* < 0.001, coefficient = -10.95, 95% CI (-15.95) – (-5.95)], who rarely [*p* = 0.016, coefficient = -6.62, 95% CI (-12.00) – (-1.24)], and who often considered quitting studying [*p* = 0.011, coefficient = -7.08, 95% CI (-12.49) – (-1.67)] had lower scores compared to those who always considered quit studying. Last, participants who were deprived of sufficient sleep (*p* = 0.029, coefficient = 3.93, 95% CI 0.42 – 7.44) had higher anxiety levels than interns who got enough sleep.

## Discussion

According to a narrative review of studies between 1990 and 2015, 35%-55% of medical students suffered emotional exhaustion, depersonalization, and other burnout-related symptoms [37], supporting the studies unveiling that more than half of medical students experienced burnout [1, 16]. Furthermore, in a more recent study, the frequency of burnout syndrome among university students was estimated to be 55.4% for emotional weariness, 31.6% for cynicism, and 30.0% for academic efficacy [38]. Depression possibly came with underlying burnout, leading to more severe depression [23, 39, 40]. Previously reported depression rates in medical students seemed to be depending on the sample, measured at as high as 51.3% in one medical school in India, while it was 23.5% among German medical students [41, 42]. According to a study, the medical student anxiety level was at the 85^th^ percentile compared to the age-matched peers in the general population [14, 43]. Severe anxiety mainly arose from medical licensing examinations [44]. One study found that 22% of medical students experienced "moderately high" to "extremely high" test anxiety [45].

Most studies in the literature of medical student mental wellness revolved around the medical students who prepared for USMLE. However, their results should not be generalized to the medical students preparing for different exams all around the world. This study is a step to relieve the need for new samples since our participants were medical students preparing for the ESM in Turkey. Thus, our research not only elucidates the link between medical student general psychological distress and the preparation period for the specialty exam in Turkey but also helps to understand whether the results are comparable to previous studies with different samples.

Our study found that the following variables are associated with final-year medical student mental wellness: attendance to preparatory courses, duration of preparation, consideration of quitting studying, and psychiatric drug usage/ongoing psychotherapy.

Most final-year medical students in Turkey wish to prepare for the ESM. According to a study, almost all final-year medical students attended commercial test preparation courses for the ESM [32]. In other words, final-year medical students wanted to allocate more time and effort to prepare for the ESM. It was consistent with our findings which demonstrate that more than half of the students considered their studies insufficient. Although most students attended preparatory courses for higher ESM scores, corresponding US studies showed that educational advancement provided by these courses was minimal or absent [46]. Despite this notorious fact, most students still opt for attending preparatory courses due to the anxiety associated with medical examinations in the US [47, 48]. Consistent with this data, attending preparatory courses for ESM was associated with poor mental wellness, as those who attended preparatory courses had higher scores on the depression subscale than those who did not. The first explanation is that medical students with psychological problems during preparation tended to attend preparatory courses rather than work alone. On the other hand, considering the hectic pace of the preparatory courses, medical students who attended these courses may experience psychological problems due to the extra stress burden they undertook after attending these courses.

In our study, students who spent less than 6 months preparing for ESM had higher stress levels than those who spent 1–2 years for the preparation. One reasonable explanation for that is insecurity. Insecurity caused by insufficient preparation might have led to elevated stress levels. On the other hand, our study revealed that stress levels were similar for medical students who considered their studies insufficient and those who did not. However, the term "insufficient" is controversial since some students are perfectionists and others are easily satisfiable. Hence, medical students who considered their studies insufficient did not necessarily spend less time studying than those who did not. Feeling insufficient to pass the specialty exam may result in elevated stress, whereas feeling insufficient to acquire the best positions may not relate to stress levels. Further studies should investigate the role of insecurity caused by insufficient preparation on medical student stress.

Final-year medical students who considered quitting studying got higher scores on all subscales. First, depressed interns may not have had enough motivation to study anymore since depression is known to cause reluctance. Second, medical students who did not trust their medical skills may have considered quitting studying due to anxiety. In addition, high anxiety exacerbated by harsh conditions and low salaries may have resulted in quitting studying for the ESM and looking for other opportunities. For example, most medical students (73.27%) reported that they would like to work abroad in the future. Lastly, interns who could not take the psychological burden caused by the high-stress environment might have tended to consider quitting studying more. In short, final-year medical students with higher depression, anxiety, and stress levels considered quitting studying more often than others.

In our study, we discovered that 34.65% of students used prescription psychiatric drugs or received psychotherapy. Furthermore, according to multivariate analysis, medical students who used prescription psychiatric drugs or received psychotherapy while preparing for ESM had higher anxiety subscale scores than those who did not. According to Ministry of Health statistics, antidepressant use in Turkey in 2020 was 49 per 1000 individuals [49]. Although we have no data on the number of individuals receiving psychotherapy in Turkey, it is safe to say that antidepressant use is more prevalent among our cohort than among the general population. Moreover, Fasanella et al. found that the prevalence of psychotropic drug use among first- to final-year medical school students at a private university in Brazil was 30.4% [50]. In a separate study, 2.4% of 4345 medical students from 35 French medical schools reported using antidepressants, 5.4% reported using anxiolytics, and 6.5% reported psychiatric or psychotherapeutic follow-up [51]. Consequently, the high rate of antidepressant consumption and psychotherapeutic follow-up in our cohort may be explained by ESM preparation, which is further supported by another study indicating a gradual decline in mental health during medical school [29]. Further research is required to establish the link between ESM preparation and the high rate of antidepressant consumption and psychotherapeutic follow-up, specifically by assessing the antidepressant consumption and psychotherapeutic follow-up of doctors in residency training in Turkey.

In our study, demographic variables and general psychological distress were not correlated. To conduct a detailed statistical analysis, we assessed the impact of GPA on mental wellness and found no correlation. Being mindful of nutrition and setting aside time for exercising were demonstrated to be irrelevant to mental wellness, but those who could not get enough sleep had higher stress levels. Although almost half of the students started using or increased their consumption of cigarettes, alcohol, or drugs and could not set aside time for themselves and their families or friends, these variables were not related to poor mental wellness. Surprisingly, final-year medical students who believed they would not get the worth of their study had similar results on all subscales to those who believed they would.

### Limitations

Our study is not without limitations. First, the response rate among the targeted cohort was low (21.35%). The medical school from which the sample was derived is one of the top medical schools in Turkey. Therefore, the participants' lifestyles, preferences, and priorities might have been associated with the characteristics of high achievers. Further studies with samples from different schools would help to compare the data and evaluate the accuracy of our study and the generality of our conclusions. Data were collected with a questionnaire based on the interns' self-declarations. There was no external validation regarding the variables such as GPA, prescription psychiatric drugs, or prescription stimulants. In addition, although we attempted to evaluate the mental wellness of the students, we were only able to assess their depression, anxiety, and stress level, which are important components of mental wellness but not the entirety of it.

In this study, we only included final-year medical students. However, most medical students usually start to prepare for the ESM in their earlier years. Further studies may include both 5th-year and final-year medical students. Lastly, this study was conducted in an environment where the effects of the COVID-19 pandemic were still on.

## Conclusion

In Turkey, medical students prepare for the ESM in a high-stress environment. Most of them may become more depressed, anxious, and stressed during the preparation. Our survey assessed final-year medical student general psychological distress during the preparation of ESM. We found four variables were associated with mental wellness: attendance to preparatory courses, duration of preparation, consideration of quitting studying, and psychiatric drug usage/ongoing psychotherapy. With its distinguished sample, this study will be an excellent source for researchers and authorities who aim to improve medical student mental wellness.

## Data Availability

The datasets generated and/or analysed during the current study are not publicly available due to them containing information that could compromise research participant privacy/consent but are available from the corresponding author on reasonable request.
